# Rotavirus Gastroenteritis Infection Among Children Vaccinated and Unvaccinated With Rotavirus Vaccine in Southern China: A Population-Based Assessment

**DOI:** 10.1001/jamanetworkopen.2018.1382

**Published:** 2018-08-31

**Authors:** Chuanxi Fu, Zhiqiang Dong, Jichuan Shen, Zhicong Yang, Ying Liao, Wensui Hu, Sen Pei, Jeffrey Shaman

**Affiliations:** 1 School of Public Health, Zhejiang Chinese Medical University, Hangzhou, China; 2 Guangzhou Center for Disease Control and Prevention, Guangzhou, China; 3 Department of Environmental Health Sciences, Mailman School of Public Health, Columbia University, New York, New York

## Abstract

**Importance:**

Since 2000, the Lanzhou lamb rotavirus vaccine has been exclusively licensed in China for voluntary rotavirus gastroenteritis (RV-GE) prevention.

**Objective:**

To evaluate the association of the Lanzhou lamb rotavirus vaccination with RV-GE among children in southern China.

**Design, Setting, and Participants:**

This cross-sectional, ecological study was set in Guangzhou, China. Participants were infants possibly vaccinated (aged 2 months to 3 years) and the children ineligible for vaccination (aged ≥4 years). The study was conducted from May 1, 2007, to April 30, 2016, and the data analysis was conducted in July 2016.

**Main Outcomes and Measures:**

Annual median age at onset of RV-GE and seasonal distribution of incidence. Cases of RV-GE in Guangzhou, China, diagnosed from May 1, 2007, to April 30, 2016, and reported to the National Information System for Disease Control and Prevention were examined. Poisson regression models were fitted among 32 452 children younger than 4 years and among 450 children who had been ineligible for vaccination, while controlling for secular trends, socioeconomic status, and meteorological factors. Logistic regression was used to assess the indirect effects provided by the vaccinated infants from 2009 to 2011 on unvaccinated infants aged 2 to 35 months based on a separate case-control data set.

**Results:**

During 9 seasons, 119 705 patients with gastroenteritis were reported; 33 407 were confirmed for RV-GE (21 202 [63.5%] male, 32 022 [95.8%] aged <4 years, and 31 306 [93.8%] residing in urban districts). The median age at onset for all patients with RV-GE increased from 11 months during the 2007 season to 15 months during the 2015 season, and the onset, peak, and cessation of incidence were delayed. When citywide vaccination coverage in the prior 12 months was classified into high and low groups (≥8.36% vs <8.36%), the incidence rate ratio for the high coverage group decreased by 32.4% among children younger than 4 years (incidence rate ratio, 0.676; 95% CI, 0.659-0.693; *P* < .001). Among the children ineligible for vaccination, the incidence rate ratio in higher coverage periods was 0.790 (95% CI, 0.351-0.915; *P* < .001) compared with the lower coverage. Compared with districts with 14% or less vaccination coverage, the adjusted odds ratio for RV-GE among unvaccinated children younger than 3 years was 0.85 (95% CI, 0.73-0.99; *P* = .03) for districts with 15% to 19% of coverage, and 0.79 (95% CI, 0.67-0.93; *P* = .004) for districts with more than 20% of coverage.

**Conclusions and Relevance:**

This study provides evidence of the population health benefits of the Lanzhou lamb rotavirus vaccination in preventing RV-GE among children in China younger than 4 years, including herd effects.

## Key Points

**Question:**

What is the effect of the Lanzhou lamb rotavirus vaccination?

**Findings:**

In this cross-sectional, ecological study of 33 407 patients with rotavirus gastroenteritis from 2007 to 2015 seasons in China, vaccination was associated with a 4-month increase in median age at onset and with delays in onset, peak, and cessation of incidence. The incidence rate ratio among children younger than 4 years and among children ineligible for vaccination decreased as citywide vaccination coverage increased, and the adjusted odds ratio for rotavirus gastroenteritis among unvaccinated infants decreased in areas with higher vaccination coverage.

**Meaning:**

The Lanzhou lamb rotavirus vaccination can provide population health benefits in preventing rotavirus gastroenteritis, including herd effects.

## Teaser

This cross-sectional, ecological study examines the age at onset and seasonal distribution of rotavirus gastroenteritis incidence among children in China vaccinated with the Lanzhou lamb rotavirus vaccine vs those ineligible for vaccination.

## Introduction

Rotavirus is the most common cause of severe diarrhea among children younger than 5 years globally. Before the introduction of the rotavirus vaccination, nearly every infant was infected with rotavirus at least once.^1,2,3,4^ Estimates for children younger than 5 years indicate that rotavirus was responsible for 528 000 (range, 465 000-591 000) deaths in 2000 and 215 000 (range, 197 000-233 000) in 2013.^5^ In 2009, the World Health Organization Strategic Advisory Group of Experts recommended worldwide inclusion of the rotavirus vaccine in national immunization programs (NIPs), particularly in African and Asian countries with high diarrhea-related child mortality. As of March 2018, 95 countries have introduced rotavirus vaccine into their NIP and 23 countries have announced plans to introduce rotavirus vaccine into their NIP.^6^

In China, it was determined that among children younger than 5 years, rotavirus caused 42.6% of all hospitalizations for severe gastroenteritis, 32.5% of outpatient visits for diarrhea, and 9.3% of all diarrhea episodes in community settings; the health consequences of rotavirus infection were estimated to cost nearly $61.4 million per year.^7,8^ Only the Lanzhou lamb rotavirus (LLR) vaccine (Lanzhou Institute of Biological Products Co, Ltd), consisting of the monovalent serotype (G[10] P[12]) group A,^9^ was licensed and has been available since 2000 in China. One dose of the live, orally administered vaccine is recommended annually for children aged 2 months to 3 years.

The number of LLR vaccine doses produced and administered in China has been increasing^10^ (eFigure 1 in the Supplement). However, vaccine use is not geographically uniform; for example, during 2013 to 2015 over one-tenth of the country’s rotavirus vaccine was administered in Guangzhou, China (eFigure 2 in the Supplement).

Rotavirus vaccination has not been included in China’s NIP; consequently, guardians have to pay out of pocket for vaccination and the LLR vaccination coverage is low.^11^ Prior findings indicate a vaccine effectiveness (VE) of 44.3% (95% CI, 28.4%-56.7%) for 1 dose of the LLR vaccine among children aged 9 to 11 months^12^; however, beyond this study, the impact of the LLR vaccination, including changes to disease epidemiology (age at onset, seasonality, and incidence) as well as benefits to unvaccinated individuals (ie, herd immunity) remain unstudied.

We examined the association between vaccination coverage and rotavirus gastroenteritis (RV-GE) in Guangzhou, China, from May 1, 2007, to April 30, 2016. We aimed to evaluate the effects of LLR vaccination on population health, including any indirect impact to unvaccinated individuals. Specifically, we examined changes in median age at onset RV-GE, onset timing, and indirect protection as a function of time and vaccination coverage.

## Methods

### Data Sources

Guangzhou lies on the Tropic of Cancer, with a subtropical climate and a population density of 1889 persons per km^2^ as of 2016 (7434.40 km^2^ and 14.04 million permanent population). Guangzhou is the largest trading city and the central city of southern China. The city consists of 10 urban districts and 2 rural districts.

For this study, we obtained data from 5 sources. The first data source provided a time series of RV-GE incidence. Medical practitioners in Guangzhou have been required to report infectious acute gastroenteritis (AGE) cases, including RV-GE, to the National Information System for Disease Control and Prevention, a physician-based system for reporting all suspected cases of infectious disease. Patients with RV-GE are defined as presenting with watery vomiting or watery diarrhea with feces testing positive for rotavirus by an enzyme-linked immunosorbent assay (RIDASCREEN Rotavirus; R-Biopharm AG). We defined the year-long rotavirus season as May through April of the following year and restricted this study to 9 RV-GE seasons spanning from May 1, 2007 to April 30, 2016.

The second source provided vaccination data, which were retrieved from the Guangzhou Children’s Expanded Programmed Immunization Administrative Computerized System. This system was designed to manage children’s vaccination information.^13^ We used the annual permanent population of children younger than 4 years in Guangzhou from the Bureau of Statistics of China as the denominator when calculating vaccination coverage.

The third source was published data from a case-control study of vaccine effectiveness during 2009 through 2011 in Guangzhou in which the rotavirus vaccination record for each child was confirmed.^12^ The fourth data set provided information on citywide demographics and socioeconomic status from the Guangzhou Statistics Bureau. Monthly meteorological data, including maximum, mean, and minimum temperature, relative humidity, atmospheric pressure, rain, and duration of sunshine, were obtained from the fifth data source, the China Meteorological Administration.

The Guangzhou Center for Disease Control and Prevention ethics committee reviewed and approved this protocol. Informed consent was not required as the study was based on electronic systems citywide and all data presented were deidentified. This study followed the Strengthening the Reporting of Observational Studies in Epidemiology (STROBE) reporting guideline.

### Age at Onset

The median age at onset and 95% confidence interval for patients with RV-GE were computed for children aged 0 to 3 years (ie, <4 years), children aged 0 to 1 year (<24 months), all ages, and persons aged 4 years or older. We also compared the proportions of overall cases for the age groups 0 to 1 year, 2 to 3 years, and 4 years or older in the 9 seasons from 2007 to 2015, and *P* values for trends over time were obtained.

### Seasonal Onset Timing

Because the number of LLR vaccine doses administered in Guangzhou increased substantially from 2011 to 2012 (eFigure 3 in the Supplement), we examined differences in RV-GE onset for the period prior to May 2011 and the period after April 2013. For a given age grouping, the weekly percentage of RV-GE was calculated by dividing the weekly number of cases with the annual total. We set a 2% threshold for onset of the rotavirus season, ie, the onset of a rotavirus season was defined as the first of 2 consecutive weeks for which the percentage was 2% or greater. The season peak was defined as the week with the highest percentage. The end of the rotavirus season was defined as the last of 2 consecutive weeks with a proportion of cases 2% or greater. We also examined the weekly percentage of all AGE cases that were diagnosed as RV-GE to characterize the seasonality during the 2 periods.

### Association of Rotavirus Vaccination Coverage With RV-GE

We used Poisson regression to quantify the association between citywide vaccination coverage and monthly counts of patients with RV-GE , in which the total effect among children aged 2 months to 3 years and the indirect effect among the children ineligible for vaccination (aged ≥4 years at the start of vaccination) were evaluated. Secular trends (using a sequential numeric variable for study year), citywide demographics, and socioeconomic status including population density (persons/per meter squared in urban and rural districts), Engels coefficient (percentage), and per capita public green area (meters squared per person) were controlled in the models. As meteorological factors have been reported to relate to the incidence of RV-GE,^14,15,16,17^ we also controlled for these factors in the models. Based on the Akaike information criterion estimation of goodness of fit for 0-, 1-, and 2-week lags, meteorological factors with no lag, including mean minimum temperature, relative humidity, atmospheric pressure, and duration of sunshine, were finally incorporated in the models.

To determine which of the prior vaccination periods, 3, 6, 9, 12, 24, and 36 months, was most significantly associated with changes in RV-GE incidence, we fit separate models for each of these periods (eAppendix 1 in the Supplement). To quantify incidence rate ratio (IRR), median monthly citywide vaccination coverage was used as a cut-off value and categorical variable in the models. Subsequently, models were fitted separately by sex, period, age group, and district (rural or urban) to determine if vaccination impact differed by these characteristics.

### Indirect Protection Among Unvaccinated Children Younger Than 3 Years

Based on the data set of 3588 patients with RV-GE and 3121 controls for which each patient’s rotavirus vaccination status was confirmed, we used logistic regression models to examine the relationship between districtwide vaccination coverage and diagnosis of rotavirus illness in children younger than 3 years. The dependent variable was diagnosis of RV-GE, and the independent variable was the coverage rate among different districts in Guangzhou. Covariates included sex, district, onset year and month, socioeconomic status, and meteorological factors. We hypothesized that a lower adjusted odds ratio (AOR) would be observed in districts with higher vaccination coverage.

### Sensitivity Analysis

We used 2 analyses to address potential biases. First, because many samples from the patients with AGE had been assayed for rotavirus and adenovirus simultaneously since 2011, we calculated the annual median age at onset for adenovirus diarrhea from May 2011 to April 2015 among children younger than 4 years and posited there would not be a trend similar to that observed for RV-GE. Second, we analyzed the sensitivity of the results to the alternate illness definitions of adenovirus diarrhea and unspecified AGE during May 2011 to April 2016; as for the RV-GE models, we controlled for other variables and explored the association between vaccination coverage and disease. No significant association for adenovirus and a weaker association for unspecified AGE than RV-GE were expected.

### Statistical Analysis

Age at onset in months for patients with RV-GE, AGE, and adenovirus-associated diarrhea were reported as the median and 95% confidence interval where appropriate. Analysis of variance was used to validate the trend for age at onset in consecutive years. We used Poisson regression to quantify the association between citywide vaccination coverage and monthly counts of patients with RV-GE, adenovirus-associated diarrhea, and unspecified AGE separately, controlling for potential confounding variables. Unconditional logistic regression was used to evaluate the indirect effects provided by the vaccinated children. Data analysis was conducted using IBM SPSS Statistics software, version 25 (IBM Corp). For all analyses, tests were 2-sided and *P* values less than .05 were regarded as significant.

## Results

From May 1, 2007, through April 30, 2016, there were 119 705 reported patients with AGE, of whom 33 407 patients with RV-GE were reported in Guangzhou. Most of the patients with rotavirus (32 022 [95.8%]) were younger than 4 years, 21 202 (63.5%) were male, and 31 306 (93.8%) lived in urban districts (Table 1; eAppendix 2 in the Supplement).

**Table 1.** Summary Statistics for Infectious Diarrhea for All Causes and RV-GE

Characteristic	No. (%)	
	Infectious Diarrhea for All Causes (n = 119 705)^a^	RV-GE (n = 33 407)
Sex		
Male	73 311 (61.2)	21 202 (63.5)
Female	46 394 (38.8)	12 205 (36.5)
Age, y		
0-1	82 480 (68.9)	27 869 (83.4)
2-3	10 923 (9.1)	4153 (12.4)
≥4	26 302 (22.0)	1385 (4.1)
Residence district^b^		
Urban	108 378 (90.6)	31 306 (93.8)
Rural	11 201 (9.4)	2054 (6.2)
Season		
2007	13 307 (11.1)	2807 (8.4)
2008	14 543 (12.1)	3790 (11.3)
2009	9314 (7.8)	1139 (3.4)
2010	13 472 (11.3)	2043 (6.1)
2011	14 752 (12.3)	2628 (7.9)
2012	13 631 (11.4)	3701 (11.1)
2013	15 926 (13.3)	7605 (22.8)
2014	13 350 (11.2)	5506 (16.5)
2015	11 410 (9.5)	4188 (12.5)
2007-2010	13 390 (50.0)	2425 (30.6)
2013-2015	13 350 (49.9)	5506 (69.4)

Abbreviation: RV-GE, rotavirus gastroenteritis.

^a^ Here, infectious diarrhea does not include cholera, dysentery, typhoid and paratyphoid.

^b^ For the variable of residence district, information for 126 patients with infectious diarrhea and 47 patients with RV-GE were missing.

### Age at Onset

**Figure 1.**
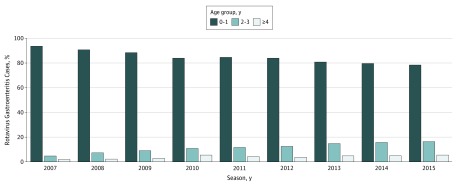
Age Group Proportion for Patients With Rotavirus Gastroenteritis, 2007-2015 Seasons

Across all cases, median age at onset for RV-GE increased from 11 (95% CI, 10.9-11.5) months in 2007 to 12 (95% CI, 11.9-12.6) months in 2011, 13 (95% CI, 12.6-13.2) months in 2012, 14 (95% CI, 13.9-14.4) months in 2013, and 15.4 (95% CI, 15.1-15.7) months in 2015, an increase of 4 months in 9 seasons (eFigure 4 in the Supplement). For the periods 2007 to 2010 and 2013 to 2015, the median age at onset was 11.84 (95% CI, 11.80-11.84) months and 14.49 (95% CI, 14.33-14.63) months, respectively. A similar trend was also found among the age groups younger than 4 years and younger than 2 years but was not detected among children older than 4 years. A shift in the annual proportion of reported patients with RV-GE for age groups younger than 2 years was observed (Figure 1). The proportion of cases for children younger than 2 years decreased 15.1% from 93% in 2007, 84% in 2011, and to 78% in 2015. At the same time, the proportion of cases among children 2 to 3 years increased 11.6% from 4.7% in 2007 to 16.3% in 2015, and among children older than 4 years increased by 3.5% from 1.9% in 2007 to 5.4% in 2015. When the 2007 to 2010 and 2013 to 2015 seasons are combined, the proportion of cases decreased by 10% among children younger 2 years and increased by 7.9% for children aged 2 to 3 years and 2.1% for children older than 4 years.

### Seasonal Onset Timing

**Figure 2.**
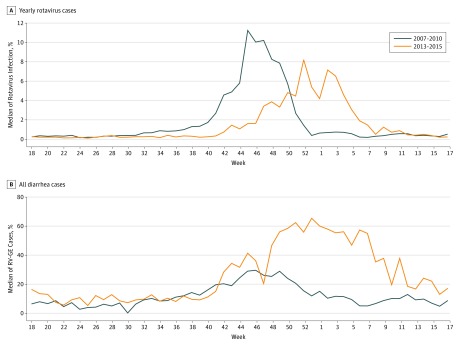
Weekly Percentage of Annual Rotavirus Gastroenteritis for 2007-2010 Seasons and 2013-2015 Seasons

During 2013 to 2015, the median onset of RV-GE occurred in mid-November (week 47), a delay of 6 weeks from the 2007 to 2010 seasons, during which onset occurred in early October (week 41) (Figure 2A). The median peak in RV-GE during the 2007 to 2010 period occurred in early November (week 45), with 11.3% of the annual total of patients with rotavirus. For the 2013 to 2015 period, RV-GE peaked in week 52 at 8.2%, a 7-week delay and 27% decrease from the prior period. The median end of the rotavirus season during the 2007 to 2010 period occurred in late December (week 52) and the end during the 2013 to 2015 period was found in week 6. Similar changes in timing were also observed for the percentage of weekly RV-GE per weekly AGE, and a higher percentage of this quantity was noted for much of the 2013 to 2015 seasons (week 42 through the following week 20) (Figure 2B; eFigure 5 in the Supplement).

### Association of Vaccination Coverage With RV-GE

We selected the median citywide vaccination coverage in the prior 12 months for further analysis among children younger than 4 years, as it showed the most significant effect on disease (eFigure 6, eFigure 7, and eFigure 8 in the Supplement). Citywide, median vaccination coverage during the prior 12 months was 8.36%; this quantity was used as the cut-off point to classify high and low vaccination coverage. The IRR for RV-GE among children younger than 4 years was 0.676 (95% CI 0.659-0.693) for high coverage districts (≥8.36%) compared with low (<8.36%) vaccination coverage, based on 32 452 patients with RV-GE. This finding indicates higher vaccination coverage (≥8.36%) and decreased in patients with RV-GE (32.4%) compared with lower coverage. A similar IRR was found among males and female, and among children aged 0, 1, and 2 to 3 year. The IRR was stronger in the urban districts than in the rural districts (Table 2).

**Table 2.** Rotavirus Gastroenteritis IRR Among Infants in High Vaccination Coverage Districts (≥8.36%) Compared With Low Coverage (<8.36%) During the Prior 12 Months Among Children Aged 0 to 3 Years Old, 2007 to 2015 Seasons^a^^,^^b^^,^^c^

Characteristic	IRR (95% CI)	*P* Value
Age, y		
0	0.673 (0.647-0.699)	<.001
1	0.694 (0.667-0.722)	<.001
2-3	0.640 (0.600-0.683)	<.001
Period		
2007-2010	1.093 (1.001-1.190)	.05
2011-2012	0.64 (0.491-0.830)	.001
2013-2015	0.33 (0.308-0.350)	<.001
Sex		
Male	0.679 (0.658-0.700)	<.001
Female	0.673 (0.646-0.702)	<.001
District^d^		
Rural	0.826 (0.749-0.910)	<.001
Urban	0.728 (0.709-0.747)	<.001
Total	0.676 (0.659-0.693)	<.001

Abbreviation: IRR, incidence rate ratio.

^a^ The median (8.36%) of vaccination coverage during the prior 12 months was used as the cut-off point to classify high and low vaccination coverage. Regressions were fitted to quantify the association between monthly citywide vaccination coverage and monthly counts of patients with rotavirus gastroenteritis.

^b^ Secular trends, variation for laboratory confirmation, population density, Engels coefficient, per capita public green areas, and the meteorological factors including the minimum temperature, relative humidity, atmospheric pressure, and duration of sunshine were controlled in the models. Each Poisson model was fitted separately for age group, period, sex, and district. Population size was accounted for in the models as an offset.

^c^ Models were fitted separately by sex, period, age group, and district (rural or urban).

^d^ The IRRs for residence district were evaluated based on the district (rural or urban), vaccination coverage, and population density.

Of the 533 patients with RV-GE among children too old to have been vaccinated, 450 (85%) were reported during the 2010 to 2015 seasons. When vaccination coverage during the prior 12 months was used, the IRR for the higher coverage bracket was 0.790 (95% CI, 0.351-0.915; *P* < .001) compared with the lower.

### Unvaccinated Infants Younger Than 3 Years

The citywide LLR vaccination coverage among patients was estimated to be 16.4% (95% CI, 15.2%-17.6%) and ranged from 6.8% to 28.6% among the 12 districts during the 2009 to 2011 period. We categorized districtwide vaccination coverage as 14% or less, 15% to 19%, and 20% or greater, based on 5% increments that most closely defined terciles. The AORs for RV-GE in unvaccinated children younger than 3 years were then computed for districts within each category. Districts with greater vaccination coverage were associated with a lower AOR. Specifically, compared with the grouping of districts with LLR vaccination coverage 14% or less, the AOR for RV-GE among unvaccinated children younger than 3 years was 0.85 (95% CI, 0.73-0.99; *P* = .03) for districts with 15% to 19% vaccinated, and 0.79 (95% CI, 0.67-0.93; *P* = .004) for districts with coverage of 20% or greater.

### Sensitivity Analysis

No increasing trend was observed for the median age at onset among 3997 patients with adenovirus-associated diarrhea observed in children younger than 4 years during the 2011 to 2015 seasons (eFigure 9 in the Supplement). Differences in the LLR vaccination coverage during the prior 12 months produced no significant IRR for the number of patients with adenovirus-associated diarrhea among children younger than 4 years or the children ineligible for vaccination. An association of vaccination coverage with unspecified patients with AGE in children younger than 4 years and the children ineligible for vaccination was found (Table 3); however, this association was weaker for patients with RV-GE.

**Table 3.** Adenovirus-Associated Diarrhea and Unspecified AGE IRR, 2011 to 2015 Seasons^a^

Characteristic	Adenovirus-Associated Diarrhea		Unspecified AGE	
	IRR (95% CI)	*P* Value	IRR (95% CI)	*P* Value
Aged <4 y	0.939 (0.852-1.034)	.20	0.962 (0.933-0.992)	.01
Vaccination ineligible	0.643 (0.318-1.300)	.21	0.976 (0.967-0.986)	<.001

Abbreviations: AGE, acute gastroenteritis; IRR, Incidence Rate Ratio.

^a^ Poisson regression models were fitted to quantify the association between citywide vaccination coverage (≥8.36% vs <8.36%) and monthly counts of adenovirus-associated diarrhea or unspecified AGE, in which secular trends, population density, Engels coefficient, per capita public green areas, and meteorological factors were controlled.

## Discussion

Using RV-GE and vaccination data from Guangzhou, China, during 9 seasons, we found a 4-month increase of the median age at onset, a delay in the seasonal distribution of RV-GE incidence, and an inverse relationship between vaccination coverage in children younger than 4 years and RV-GE incidence in the children eligible for vaccination as well as the unvaccinated population.

Prior studies have explored the population impact of rotavirus vaccination for RotaTeq (Merck & Co) and ROTARIX (GlaxoSmithKline Biologicals), which have demonstrated good efficacy (85%-98%) in clinical trials^18,19^ and various effectiveness in different countries (eTable in the Supplement); many developed countries as well as some low-income countries in Africa and Asia have adopted the rotavirus vaccine as part of their NIPs since 2006.^20^ A systematic review reported a 49% to 89% decline in rotavirus-associated hospitalizations among children younger than 5 years within 2 years of vaccine introduction.^21^ Unexpected benefits of rotavirus vaccine were also noted.^22,23^ However, for the LLR monovalent animal strain vaccine, the impact has not been evaluated.

Countries with NIPs for rotavirus have observed dramatic reductions of infant cases.^23,24,25,26,27,28,29^ For example, in Austria, the median age of hospitalized children with RV-GE increased by 10 months from 14.3 months before 2006 to 23.4 months in 2009 after the implementation of an NIP among infants in 2007.^30^ This change is much larger than our findings (4 months) in China, where vaccination is voluntary and coverage much less comprehensive. We also observed delays of 6 weeks for RV-GE onset, 7 weeks for peak, and 6 weeks for epidemic end between 2007 to 2010 and 2013 to 2015. Tate et al^31^ reported marked changes in the timing of the rotavirus season as well as substantial reductions in rotavirus activity. However, rotavirus infection control and surveillance for rotavirus activity in Guangzhou has not been a priority and relevant data to assess changes in incidence are unavailable, although the country has a national rotavirus surveillance network; as a result, we cannot directly compare rotavirus activity among different vaccination periods and thus cannot determine whether the shift of seasonality was due to vaccination or other factors.

Higher LLR vaccination coverage was associated with a 32.4% decreased incidence risk among children younger than 4 years. In a meta-analysis of VE for the LLR vaccine, 10 prospectively designed studies during 2000 to 2012 reported VE of 72% (95% CI, 63%-79%) against RV-GE in children younger than 5 years.^32^ By combining the LLR vaccination coverage among children younger than 36 months of 46.8% (95% CI, 44.0%-49.7%) in Guangzhou in 2013^11^ with this 72% VE, we can crudely estimate a decreased incidence of 33.7% (range, 27.7%-39.3%), which is similar to the level reported in this study. The genotype of the LLR vaccine is stated to be G[10]P[12]; however, a separate analysis characterized the strain as G[10]P[15],^33^ and a cross-protective effect between vaccine strains and circulating strains has been noted.^12,34^ Vaccination benefits were seen across age groups and sex, but were most pronounced in urban districts (eAppendix 3 in the Supplement).

Several prior studies have evaluated the indirect effects of RV vaccination^35,36,37^ (eAppendix 4 in the Supplement). We evaluated the indirect impact of the LLR vaccine on children ineligible for vaccination and a decreased IRR was reported, which is much lower than in other rotavirus vaccine studies in regions with higher vaccination coverage. We also reported a decrease of one-fifth among unvaccinated children younger than 3 years in areas with higher vaccination coverage, providing evidence of benefits of rotavirus vaccination to young children. However, this protection could be derived from either natural infection or vaccination.

Two additional issues may complicate LLR vaccination effectiveness and uptake. First, timing of the current regimen, in which children receive 1 dose per year for 3 consecutive years beginning at age 2 months, may not be ideal. In China, 94.5% of infants have rotavirus-associated diarrhea during the first 2 years of life, 16.9% have rotavirus infection by age 6 months, and 59.1% have rotavirus infection by age 1 year.^38,39^ In Guangzhou, vaccine coverage was 8.2% and 40% in children younger than 1 year and younger than 2 years, respectively.^38,39^ It is essential that children receive a vaccination as soon as possible to ensure the induction of protection prior to natural rotavirus infection. Second, the rotavirus vaccine has not been included in the NIP in China, and the vaccine is priced at $63 for 2 doses, which is almost a tenth of a worker’s average monthly salary in China.^40^ As a result, uptake is low and only 10% of children received 2 or 3 doses of the LLR vaccine in Guangzhou. The vaccine has been in use for 16 years in China on a voluntary basis, but not through a more comprehensive, nationally funded program. A cost-effectiveness model has shown that in China, a 2-dose rotavirus vaccination program could annually avert 3013 deaths (62%), 194 794 hospitalizations (59%), and 1 333 356 outpatient visits (51%) associated with rotavirus disease.^8^ Free rotavirus vaccination needs to be incorporated into the NIP in China to effectively reduce deaths, hospitalizations, and outpatient visits due to rotavirus disease in China.

### Limitations

This study had limitations. We did not collect information on disease severity and thus are unable to analyze vaccination impact on case severity. Calculation of vaccination coverage in the study used the number of vaccine doses consumed as the numerator and the permanent resident population as the denominator. This calculation supposes that each infant was vaccinated with 1 dose; however, one-tenth of infants were given 2 or 3 doses in Guangzhou. Further, the permanent resident population of children younger than 4 years may be much larger than that in the vaccination system and coverage may thus be less than estimated here. Because there are no surveillance data for rotavirus activity in Guangzhou, our analysis was based on reported RV-GE data and may not be robust enough to provide insight into changes in rotavirus seasonality. Further, analyses in other areas of China are needed to validate our findings. Modeling secular trends, numbers of cases, and even vaccination coverage may be temporally correlated, which could have affected the accuracy of the presented *P* values and confidence intervals. Our results were based on cross-sectional or ecological data, which cannot confirm a causal association between vaccination coverage and the onset of disease in different areas. Future work should be performed using more rigorous designs to confirm vaccination impacts.

## Conclusions

In an analysis of 9 seasons of RV-GE data, vaccination coverage was negatively associated with the risk of RV-GE and positively associated with age at onset. These results provide evidence for the population impact of the LLR vaccination, which can be used to inform the China NIP on the possible benefits of rotavirus vaccination.

## Notes

Supplement. **eAppendix 1.** How to Choose the Most Significant Cumulative Coverage **eAppendix 2.** RV-GE Cases in 2007-2010 and 2013-15 Seasons **eAppendix 3.** Vaccination Benefits in Different Populations **eAppendix 4.** Studies Which Have Evaluated the Indirect Effects of RV Vaccination **eFigure 1.** The Number of LLR Vaccine Doses Manufactured and Annual Reported Infectious Diarrhea Cases in China, 2002-2015 **eFigure 2.** Annual Rotavirus Vaccine Consumption in Eight Cities in China, 2000-2015 **eFigure 3.** Incidence Rate of Infectious Diarrhea, Rotavirus Gastroenteritis and Cumulative Rotavirus Vaccination Coverage In Guangzhou, 2005-2015 **eFigure 4.** Median Onset Age (value and 95% CI) for Rotavirus Gastroenteritis During 2007-2015 **eFigure 5.** The Weekly Number of RV-AGE and AGE Cases in Guangzhou During May 2007 Through April 2016 **eFigure 6.** The Actual and Fitted Number of Rotavirus Cases of <4 Years Old, During May 2007 to April 2016 **eFigure 7.** Time Series of LLR Vaccination Coverage During the Prior 3, 6, 9, 12, 24, and 36 Months in Guangzhou, 2007 to 2015 Seasons **eFigure 8.** Incidence Rate Ratios (IRRs) for Monthly RV-GE Cases As a Function of Different Prior Vaccination Periods **eFigure 9.** Median Onset Age (95% CI) for Adenovirus Diarrhea Aged 0-3 Years in Guangzhou, 2011-2015 **eTable.** Median RV1 Vaccine Effectiveness (VE) and 95% Confidence Intervals in Countries With Different Under-5 Child Mortality Rates **eReferences**
