# Supplementary material for: Rotavirus Gastroenteritis Infection Among Children Vaccinated and Unvaccinated With Rotavirus Vaccine in Southern China: A Population-Based Assessment
Source: JAMA Netw Open. 2018 Aug 31;1(4):e181382. doi: 10.1001/jamanetworkopen.2018.1382 (PMC6324266; doi:10.1001/jamanetworkopen.2018.1382)
Supplement: Supplement. — eAppendix 1. How to Choose the Most Significant Cumulative Coverage eAppendix 2. RV-GE Cases in 2007-2010 and 2013-15 Seasons eAppendix 3. Vaccination Benefits in Different Populations eAppendix 4. Studies Which Have Evaluated the Indirect Effects of RV Vaccination eFigure 1. The Number of LLR Vaccine Doses Manufactured and Annual Reported Infectious Diarrhea Cases in China, 2002-2015 eFigure 2. Annual Rotavirus Vaccine Consumption in Eight Cities in China, 2000-2015 eFigure 3. Incidence Rate of Infectious Diarrhea, Rotavirus Gastroenteritis and Cumulative Rotavirus Vaccination Coverage In Guangzhou, 2005-2015 eFigure 4. Median Onset Age (value and 95% CI) for Rotavirus Gastroenteritis During 2007-2015 eFigure 5. The Weekly Number of RV-AGE and AGE Cases in Guangzhou During May 2007 Through April 2016 eFigure 6. The Actual and Fitted Number of Rotavirus Cases of <4 Years Old, During May 2007 to April 2016 eFigure 7. Time Series of LLR Vaccination Coverage During the Prior 3, 6, 9, 12, 24, and 36 Months in Guangzhou, 2007 to 2015 Seasons eFigure 8. Incidence Rate Ratios (IRRs) for Monthly RV-GE Cases As a Function of Different Prior Vaccination Periods eFigure 9. Median Onset Age (95% CI) for Adenovirus Diarrhea Aged 0-3 Years in Guangzhou, 2011-2015 eTable. Median RV1 Vaccine Effectiveness (VE) and 95% Confidence Intervals in Countries With Different Under-5 Child Mortality Rates eReferences [file jamanetwopen-1-e181382-s001.pdf]

Supplementary Online Content

Fu C, Dong Z, Shen J, et al. Rotavirus gastroenteritis infection among children vaccinated and unvaccinated with rotavirus vaccine in southern China: a population-based assessment. *JAMA Netw Open*. 2018;1(4):e181382. doi:10.1001/jamanetworkopen.2018.1382

eAppendix 1. How to Choose the Most Significant Cumulative Coverage
eAppendix 2. RV-GE Cases in 2007-2010 and 2013-15 Seasons
eAppendix 3. Vaccination Benefits in Different Populations
eAppendix 4. Studies Which Have Evaluated the Indirect Effects of RV Vaccination
eFigure 1. The Number of LLR Vaccine Doses Manufactured and Annual Reported Infectious Diarrhea Cases in China, 2002-2015
eFigure 2. Annual Rotavirus Vaccine Consumption in Eight Cities in China, 2000-2015
eFigure 3. Incidence Rate of Infectious Diarrhea, Rotavirus Gastroenteritis and Cumulative Rotavirus Vaccination Coverage In Guangzhou, 2005-2015
eFigure 4. Median Onset Age (value and 95%CI) for Rotavirus Gastroenteritis During 2007-2015
eFigure 5. The Weekly Number of RV-AGE and AGE Cases in Guangzhou During May 2007 Through April 2016
eFigure 6. The Actual and Fitted Number of Rotavirus Cases of <4 Years Old, During May 2007 to April 2016
eFigure 7. Time Series of LLR Vaccination Coverage During the Prior 3, 6, 9, 12, 24, and 36 Months in Guangzhou, 2007 to 2015 Seasons
eFigure 8. Incidence Rate Ratios (IRRs) for Monthly RV-GE Cases As a Function of Different Prior Vaccination Periods
eFigure 9. Median Onset Age (95%CI) for Adenovirus Diarrhea Aged 0-3 Years in Guangzhou, 2011-2015
eTable. Median RV1 Vaccine Effectiveness (VE) and 95% Confidence Intervals in Countries With Different Under-5 Child Mortality Rates
eReferences

This supplementary material has been provided by the authors to give readers additional information about their work.

© 2018 Fu C et al. *JAMA Network Open*.

eAppendix 1. How to Choose the Most Significant Cumulative Coverage

Using district-wide electronic vaccination data for April 2004 through March 2016, we obtained the number of administered LLR vaccine doses during the prior 3, 6, 9, 12, 24 and 36 months and calculated monthly cumulative coverage from May 2007 to April 2016 among those < 4 years of age. We did not include vaccination during the current month as the rotavirus vaccine typically begins to provide protection 2 weeks after immunization.

To determine which of the prior vaccination periods, 3, 6, 9, 12, 24 and 36 months, was most significantly associated with changes in RV-GE incidence, we fit separate models for each of these periods. For use in subsequent models, we chose the prior period with the most significant Incidence Rate Ratio (IRR).

© 2018 Fu C et al. *JAMA Network Open*.

eAppendix 2. RV-GE Cases in 2007-2010 and 2013-15 Seasons

The annual number of AGE during the 2013-2015 seasons was similar to the 2007–2010 baseline; however, 5,506 cases tested positive annually for rotavirus, an increase of 127% from the baseline. Peak RV-GE cases occurred in 2008 and 2013 for the two periods respectively.

© 2018 Fu C et al. *JAMA Network Open*.

eAppendix 3. Vaccination Benefits in Different Populations

Vaccination benefits were seen across age groups and sex, but were most pronounced in urban districts and in the 2013-15 seasons. There are two possible explanations for the difference between the rural and urban districts. First, inoculation techniques, including cold chain maintenance and selection of those eligible for vaccination, may vary between the urban and rural districts. Health providers in urban districts are thought to provide better inoculation services and thus rotavirus vaccination may be more reliable than in rural areas. As a consequence, the direct impact of vaccination may be more significant in urban districts[1]. Second, as urbanization increases in China[2], population density increases in urban districts faster than in rural districts. In addition to higher urban vaccination coverage (data not shown), it is possible that social contacts at day care or community centers in urban districts provide more indirect protection to young children. However, such an association has not been evaluated.

© 2018 Fu C et al. *JAMA Network Open*.

eAppendix 4. Studies Which Have Evaluated the Indirect Effects of RV Vaccination

As for natural infection, which generates serotype-specific immunity but also cross-protection against moderate-to-severe disease due to other serotypes, vaccination with a live attenuated vaccine potentially can reduce rotavirus transmission and induce herd protection[3, 4]. For example, a dynamic transmission model projected the population-level impact of RV vaccination in children within national immunization programs in Europe. Using an assumption of 79% VE against RV-GE, the model predicted that, 5 years after implementation of a vaccination program with vaccination coverage rates of 70%, 90%, and 95%, herd protection would induce an additional reduction in RV-related GE incidence of 25%, 22%, and 20% respectively[5]. In the US, marked declines have been noted in GE in all age groups during peak rotavirus season; for example, data from the National Inpatient Sample during 2008 showed that hospitalizations due to case-unspecified GE deceased for populations up to 24 years of age[6], and a 48% decline in the prevalence of rotavirus was observed in adults who had stool sent for bacterial stool culture from 2006-2007 to 2008-2010[7]. In the United States, Lopman et al. found a remarkable 71% reduction in hospitalizations coded as rotavirus and a 30% reduction in diarrhea of unspecified causes among older children who were not vaccinated and should have been immune due to early childhood infections[6, 8].

© 2018 Fu C et al. *JAMA Network Open*.

eFigure 1. The Number of LLR Vaccine Doses Manufactured and Annual Reported Infectious Diarrhea Cases in China, 2002-2015

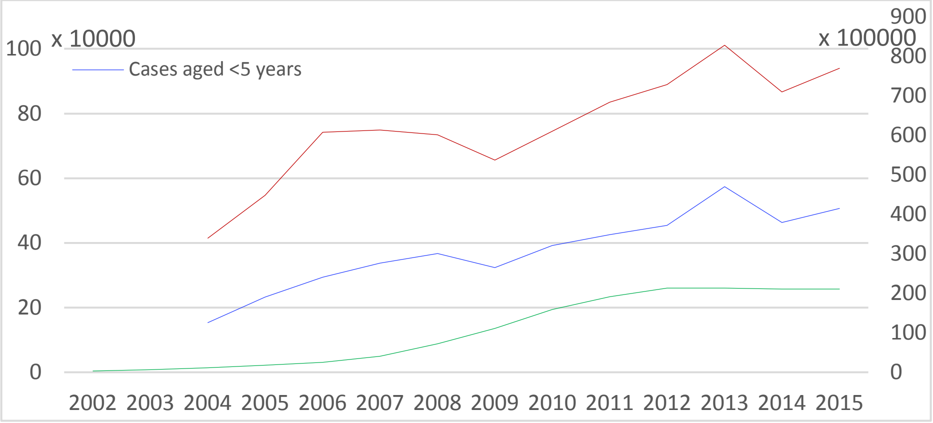

Sum of vaccine doses in the current year combined with prior two years is shown. There were 34 thousand in 2000, <1 million before 2006, and 6.9 - 7.2 million annually from 2010 to 2015.

© 2018 Fu C et al. *JAMA Network Open*.

eFigure 2. Annual Rotavirus Vaccine Consumption in Eight Cities in China, 2000-2015

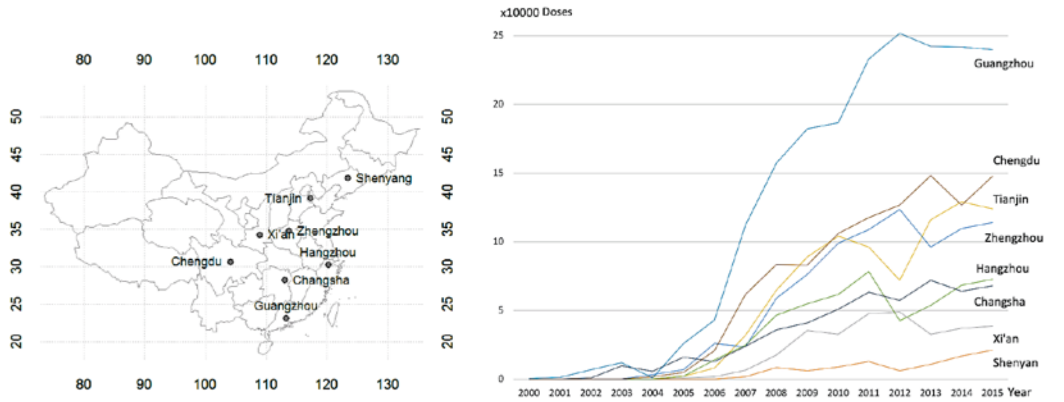

Eight cities (Shenyang, Xi'an, Tianjin, Zhengzhou, Hangzhou, Chengdu, Changsha and Guangzhou) with only 6.02% of the national population, constituted nearly one third of LLR vaccine uptake in China during 2010 to 2015 (24.5%, 27.5%, 29.9%, 32.7%, 32.8% and 33.6% each year, respectively).

Over one tenth of the country's rotavirus vaccine was administered in Guangzhou, southern China (10.5%, 10.5% and 10.2%, during 2013, 2014, and 2015, respectively).

© 2018 Fu C et al. *JAMA Network Open*.

eFigure 3. Incidence Rate of Infectious Diarrhea, Rotavirus Gastroenteritis and Cumulative Rotavirus Vaccination Coverage in Guangzhou, 2005-2015

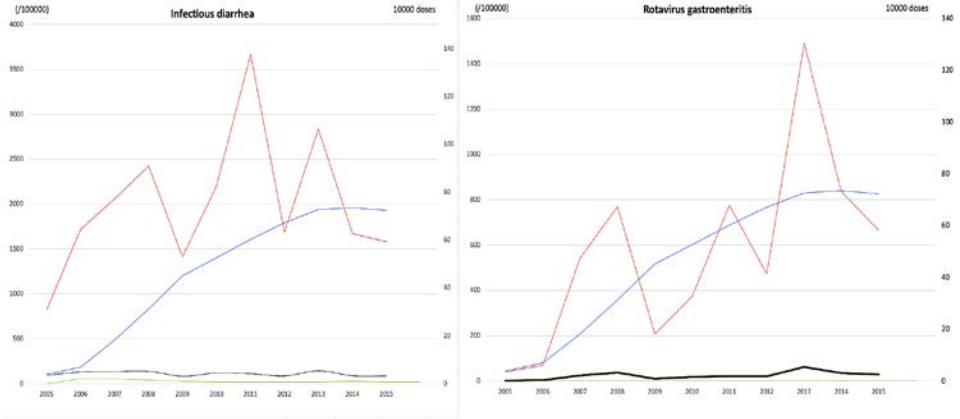

Blue line: 3-year cumulative vaccine consumption; Red line: Incidence rate for children of < 5 years old; Black: Incidence rate for all age groups; Green: incidence rate for persons of 5+ years old

© 2018 Fu C et al. *JAMA Network Open*.

eFigure 4. Median Onset Age (value and 95%CI) for Rotavirus Gastroenteritis During 2007-2015

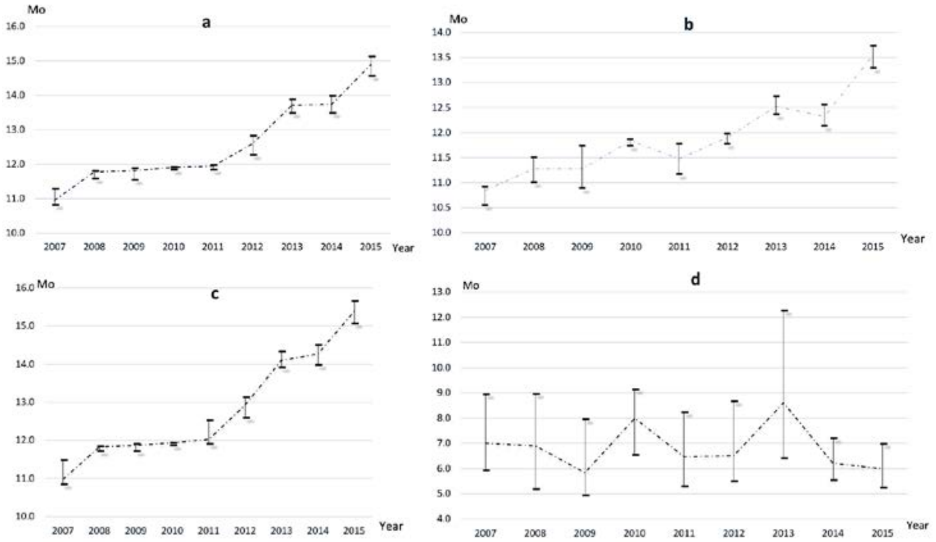

a: 0-3 years; b: 0-1 year; c: all age groups; d: 4+ years.

Linear regressions of onset age of RV-GE cases upon year, accounting for sex and district. a) RV-GE among children < 4 years old, p<0.001; b) children 0-1 year old, p<0.001; c) all ages, p<0.001; and d) persons 4+ years of age, p=0.86.

© 2018 Fu C et al. *JAMA Network Open*.

eFigure 5. The Weekly Number of RV-AGE and AGE Cases in Guangzhou During May 2007 Through April 2016

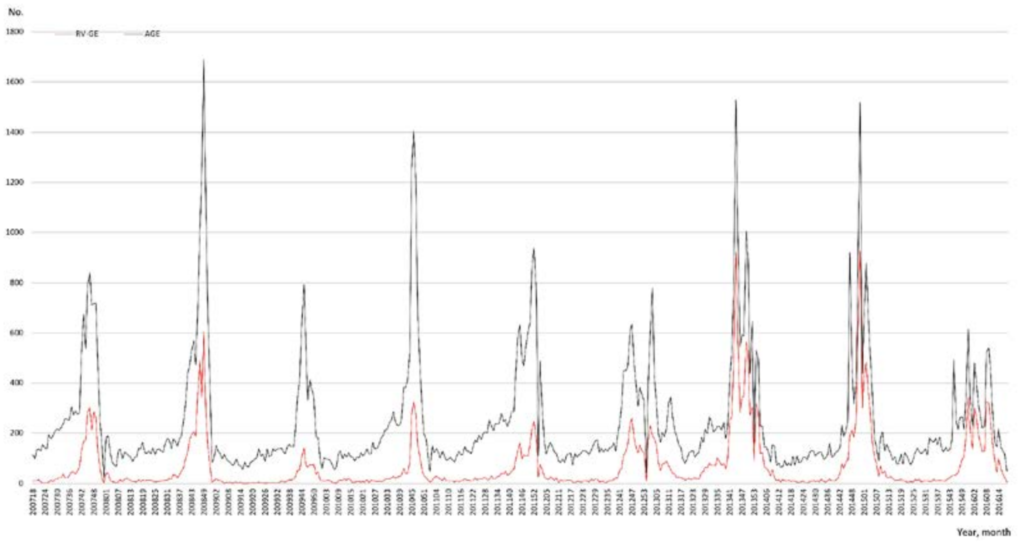

© 2018 Fu C et al. *JAMA Network Open*.

eFigure 6. The Actual and Fitted Number of Rotavirus Cases of <4 Years Old, During May 2007 to April 2016

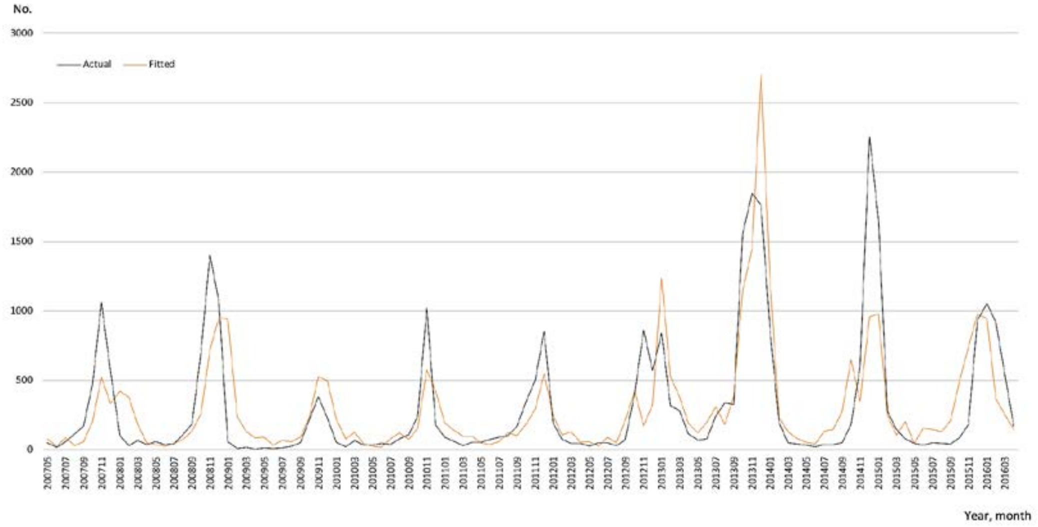

© 2018 Fu C et al. *JAMA Network Open*.

eFigure 7. Time Series of LLR Vaccination Coverage During the Prior 3, 6, 9, 12, 24, and 36 Months in Guangzhou, 2007 to 2015 Seasons

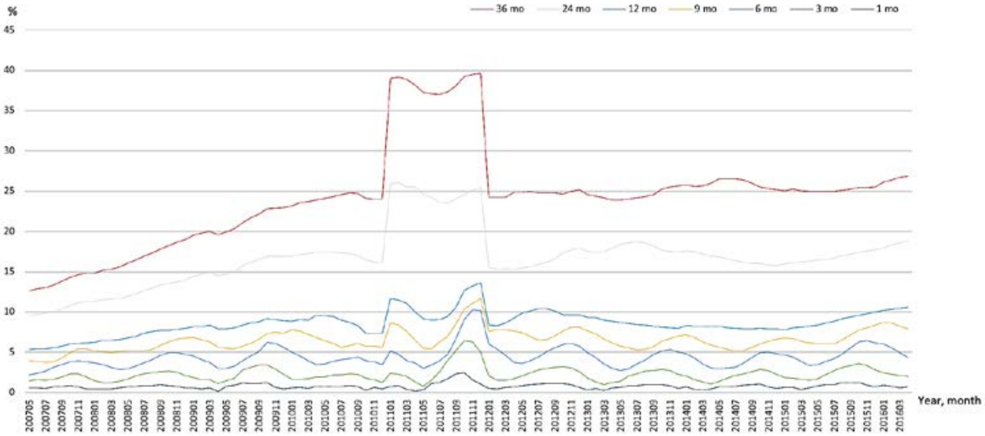

© 2018 Fu C et al. *JAMA Network Open*.

eFigure 8. Incidence Rate Ratios (IRRs) for Monthly RV-GE Cases As a Function of Different Prior Vaccination Periods

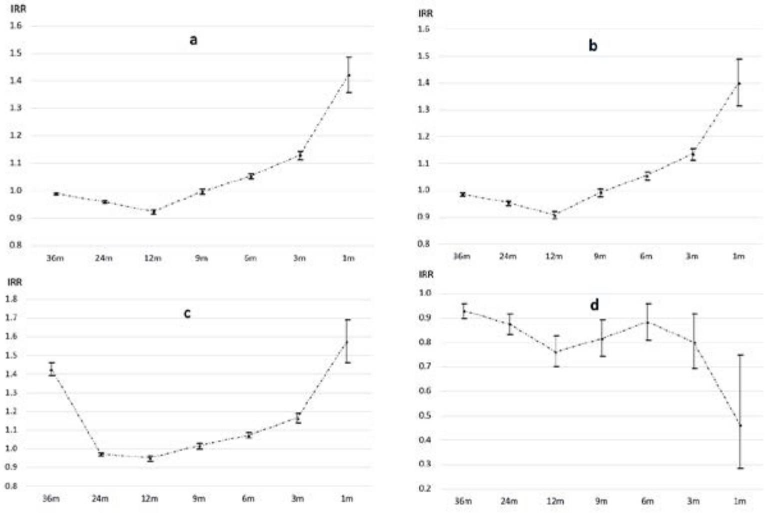

a) <4 years of age, b) <1 year, c) 12-23 months (1 year), d) Vaccination ineligible persons (>4 years at the start of rotavirus vaccination in China)

After controlling for the social, economic, meteorological and other variables, among the 6 vaccination coverage periods, coverage for the prior 12 months (IRR: 0.924, 0.915-0.932, p<0.001) was selected for further analysis among children of < 4 years of age, as it showed the most significant effect on disease.

© 2018 Fu C et al. *JAMA Network Open*.

eFigure 9. Median Onset Age (95%CI) for Adenovirus Diarrhea Aged 0-3 Years in Guangzhou, 2011-2015

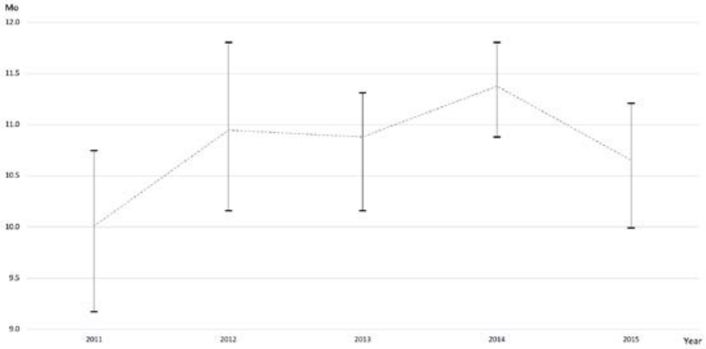

Linear regression of onset age upon year, accounting for sex and residence districts, p=0.46

© 2018 Fu C et al. *JAMA Network Open*.

eTable. Median RV1 Vaccine Effectiveness (VE) and 95% Confidence Intervals in Countries With Different Under-5 Child Mortality Rates

	Rotarix (RV1)	RotaTeq (RV5)
Low Mortality	0.82(0.72,0.88)	0.88(0.83,0.91)
Medium Motality	0.66(0.51,0.77)	-
High Mortality	0.58(0.51,0.65)	0.49(0.40,0.57)

NOTE. Low mortality countries were defined as those in the lowest quartile of under-5 child mortality rates, with medium mortality countries as those in the second quartile, and high mortality as those countries in the highest two quartiles[9].

© 2018 Fu C et al. *JAMA Network Open*.

eReferences

1. Zheng K, Yang R, Zeng W, Ye Q. Urban and Rural Primary Immunization Status in Chengdu. *Journal of Preventive Medicine Information.* 2011;27(10):786-788.

2. She X, Zhao D, Scholnick J. Measuring the Gap: A Health Assessment of Rural Chinese Children Compared to Urban Children. *Glob Pediatr Health.* 2016;3:2333794X-15625298X.

3. Velazquez FR, Matson DO, Calva JJ, et al. Rotavirus infection in infants as protection against subsequent infections. *N Engl J Med.* 1996;335(14):1022-1028.

4. Yi J, Anderson EJ. Rotavirus vaccination: short-term indirect herd protection, long-term uncertainty. *Expert Rev Vaccines.* 2013;12(6):585-587.

5. Van Effelterre T, Soriano-Gabarro M, Debrus S, Claire NE, Gray J. A mathematical model of the indirect effects of rotavirus vaccination. *Epidemiol Infect.* 2010;138(6):884-897.

6. Lopman BA, Curns AT, Yen C, Parashar UD. Infant rotavirus vaccination may provide indirect protection to older children and adults in the United States. *J Infect Dis.* 2011;204(7):980-986.

7. Anderson EJ, Shippee DB, Weinrobe MH, et al. Indirect protection of adults from rotavirus by pediatric rotavirus vaccination. *Clin Infect Dis.* 2013;56(6):755-760.

8. Glass RI. Unexpected benefits of rotavirus vaccination in the United States. *J Infect Dis.* 2011;204(7):975-977.

9. Jonesteller CL, Burnett E, Yen C, Tate JE, Parashar UD. Effectiveness of Rotavirus Vaccination: A Systematic Review of the First Decade of Global Postlicensure Data, 2006-2016. *Clin Infect Dis.* 2017;65(5):840-850.

© 2018 Fu C et al. *JAMA Network Open*.
